# Effect of Gedunin on Acute Articular Inflammation and Hypernociception in Mice

**DOI:** 10.3390/molecules20022636

**Published:** 2015-02-03

**Authors:** Fernando P. Conte, Fausto K. Ferraris, Thadeu E. M. M. Costa, Patricia Pacheco, Leonardo N. Seito, Waldiceu A. Verri, Fernando Q. Cunha, Carmen Penido, Maria G. Henriques

**Affiliations:** 1 Laboratório de Farmacologia Aplicada, Farmanguinhos, Fundação Oswaldo Cruz, Rio de Janeiro 21041-250, Brazil; E-Mails: fernando.conte@bio.fiocruz.br (F.P.C.); fausto.ferraris@incqs.fiocruz.br (F.K.F.); thadeucosta@far.fiocruz.br (T.E.M.M.C.); patricia.pacheco.silva@gmail.com (P.P.); leonardoseito@far.fiocruz.br (L.N.S.); cpenido@cdts.fiocruz.br (C.P.); 2 Departamento de Ciências Patológicas, Centro de Ciências Biológicas, Universidade Estadual de Londrina, Londrina 86057-970, Brazil; E-Mail: waverri@uel.br; 3 Departamento de Farmacologia, Faculdade de Medicina de Ribeirão Preto, Universidade de São Paulo, Ribeirão Preto 14049-900, Brazil; E-Mail: fdqcunha@fmrp.usp.br; 4 Centro de Desenvolvimento Tecnológico em Saúde, CDTS/INCT-IDN, Fundação Oswaldo Cruz, Rio de Janeiro 21045-360, Brazil

**Keywords:** zymosan, arthritis, edema, neutrophil, macrophage, lipid body, cytokine, leukotriene, prostaglandin

## Abstract

Gedunin, a natural limonoid from Meliaceae species, has been previously described as an antiinflammatory compound in experimental models of allergic inflammation. Here, we report the antiinflammatory and antinociceptive effects of gedunin in an acute model of articular inflammation induced by zymosan (500 μg/cavity; intra-articular) in C57BL/6 mice. Intraperitoneal (i.p.) pretreatment with gedunin (0.005–5 mg/kg) impaired zymosan-induced edema formation, neutrophil accumulation and hypernociception in mouse knee joints, due to decreased expression of preproET-1 mRNA and production of LTB_4_, PGE_2_, TNF-α and IL-6. Mouse post-treatment with gedunin (0.05 mg/kg; i.p.) 1 and 6 h after stimulation also impaired articular inflammation, by reverting edema formation, neutrophil accumulation and the production of lipid mediators, cytokines and endothelin. In addition, gedunin directly modulated the functions of neutrophils and macrophages *in vitro.* The pre-incubation of neutrophil with gedunin (100 µM) impaired shape change, adhesion to endothelial cells, chemotaxis and lipid body formation triggered by different stimuli. Macrophage pretreatment with gedunin impaired intracellular calcium mobilization, nitric oxide production, inducible nitric oxide synthase expression and induced the expression of the antiinflammatory chaperone heat shock protein 70. Our results demonstrate that gedunin presents remarkable antiinflammatory and anti-nociceptive effects on zymosan-induced inflamed knee joints, modulating different cell populations.

## 1. Introduction

Articular inflammation is a major clinical symptom of acute and chronic joint diseases, such as rheumatoid arthritis, osteoarthritis and gout. It is characterized by articular pain, stiffness and swelling, due to increased production of inflammatory mediators and infiltration of leukocytes into periarticular tissues, synovial membrane and cavity [1,2]. Chronification of articular inflammation can progress to erosion of cartilage and bone, causing movement limitation, disability, pain and decreased quality of life [2,3,4]. Several experimental models of articular inflammation have been described, providing tools to study the mechanisms underlying these inflammatory conditions and allowing the discovery of novel anti-inflammatory and analgesic candidates. Zymosan-induced articular inflammation is considered a model of arthritis that develops into a chronic stage in mice and rats [4,5]. It is characterized by periarticular edema, neutrophil and mononuclear cell infiltration, synovial hypertrophy, pannus formation and pain [4,5], which are mediated by the massive local generation of protein and lipid inflammatory mediators, including tumor necrosis factor (TNF)-α, interleukin (IL)-1-β, IL-6 and leukotriene (LT)B_4_ [6,7].

Neutrophils are key cells in articular inflammation. They are attracted to the tissue via the overproduction of chemoattractant mediators, namely CXCL1, CXCL2, endothelin (ET)-1 and leukotriene B_4_, a process in which resident macrophages play a central role [6,8]. Once in the tissue, activated neutrophils cause tissue damage via the release of reactive oxygen species (ROS) and proteases, such as matrix metalloproteinase (MMP)-8, MMP-9, neutrophil elastase and cathepsin G into the synovial fluid and joints [9,10,11]. In addition to their cytotoxic properties, neutrophils of immune cells contribute to the pathology of joint diseases by orchestrating the inflammatory response, modulating the functions in T lymphocytes and macrophages, via the production of chemokines and cytokines [12,13]. Indeed, decreased inflammation and joint destruction have been directly correlated with reduced neutrophil influx into the joints, as observed in mouse models by means of antibody blockade or gene deletion of chemoattractant receptors, such as CXCR1, CXCR2 and BLT1 (LTB_4_ receptor) [14,15].

Prospection of new drugs obtained from natural products (or from natural product structures) plays a compelling role in drug discovery and development [16]. Gedunin is a natural tetranortriterpenoid isolated from vegetal species of the Meliaceae family, known to inhibit the stress-induced chaperone heat shock protein (Hsp)90 [17]. Hsp90 blockade represents an attractive multi-targeted therapeutic approach, since among Hsp90 client proteins are included several kinases and transcription factors, such as NFkB [18,19]. Hsp90 inhibitors have been shown to reduce inflammatory responses in different experimental models of inflammation, including atherosclerosis, uveitis and lung inflammation [20,21,22]. In line with this, our group has previously demonstrated that a pool of six different tetranortriterpenoids, that contained gedunin, exhibited antiinflammatory effects in different experimental models, including articular inflammation [7,23].

In the current study, we demonstrate that gedunin suppresses articular inflammation in an experimental model of zymosan-induced acute articular inflammation, impairing articular neutrophil influx, edema formation, hypernociception and the production of pro-inflammatory mediators, including cytokines and lipid mediators.

## 2. Results and Discussion

### 2.1. Gedunin Pre-Treatment Impairs 6 h Zymosan-Induced Articular Edema, Neutrophil Accumulation and Hypernociception

**Figure 1:**
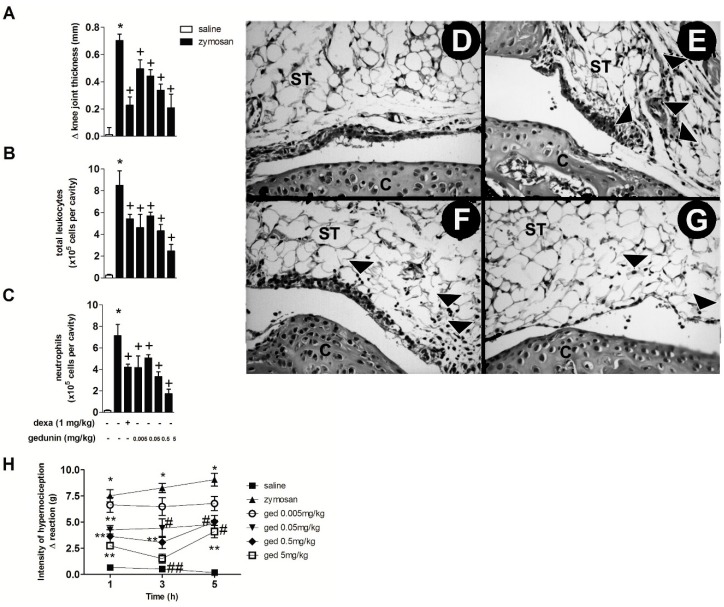
Gedunin pre-treatment inhibits zymosan-induced (**A**) edema formation, (**B**) total leukocyte and (**C**) neutrophil influx and (**H**) hyperalgesia in knee joint cavities of C57BL/6 mice within 6 h. Hematoxylin and eosin (H&E)-stained representative of histological joint sections of knee joints injected with (**D**) saline, (**E**–**G**) zymosan, (F) dexamethasone and (G) gedunin collected 6 h after stimulation. Black head arrows show inflammatory cell infiltration. C, Cartilage; ST, synovial tissue. Results are expressed as mean ± SEM. * *p* ≤ 0.05 *vs.* saline group, ^+^ *p* ≤ 0.05 *vs.* zymosan group, ^#^ *p* ≤ 0.05 *vs*. zymosan group, ** *p* ≤ 0.05 *vs*. ged 0.005 mg/kg and zymosan groups, ^##^ *p* ≤ 0.05 *vs*. ged 0.005 mg/kg, ged 0.05 mg/kg and zymosan groups.

The intra-articular (i.a.) injection of zymosan (500 μg/cavity) into mouse knee joints induced an inflammatory response within 6 h, characterized by significant edema formation and massive leukocyte accumulation, mainly due to neutrophil influx (approximately 90% of total leukocytes in synovial washes) (Figure 1A–C), as previously demonstrated [6,8]. Mouse pre-treatment with gedunin (0.005–0.5 mg/kg; i.p.) 1 h prior to i.a. zymosan stimulation significantly impaired zymosan-induced edema formation within 6 h, in a dose dependent manner (R = 0.97), achieving a maximal inhibition of 70% at the highest concentration (0.5 mg/kg). The pre-treatment with dexamethasone, used as reference antiinflammatory drug, also caused a 70% of inhibition of zymosan-induced edema formation. In addition to its anti-edematogenic effects, gedunin (0.005–0.5 mg/kg; i.p.) also reduced zymosan-induced total leukocyte influx to synovial washes within 6 h (Figure 1B), mainly due to inhibition of neutrophil migration (Figure 1C), achieving a maximal inhibition of 76% at 5 mg/kg. Dexamethasone pretreatment also inhibited zymosan-induced total leukocyte and neutrophil migration (36% and 41% of inhibition, respectively). Corroborating this data, histological analysis of knee joint tissues from zymosan-stimulated mice revealed an intense neutrophil accumulation within 6 h (Figure 1D), when compared to control group (Figure 1E), that was prevented by dexamethasone (1 mg/kg; i.p.; Figure 1F) and gedunin (0.5 mg/kg; i.p.; Figure 1G) pretreatments. Figure 1H shows that the i.a. injection of zymosan (500 μg/cavity) decreased nociceptive threshold of C57BL/6 mice when compared to control group from 1 to 5 h after injection. Gedunin i.p. pretreatment significantly reduced articular hypernociception from 0.05 to 5 mg/kg at all time-points analyzed.

The anti-edematogenic effects of *Carapa guianensis* crude oil and its fraction containing gedunin has been already described [23] and seem to be related to the inhibition of mediators such as PAF, bradykinin and PGE_2_, the latter being described as a major contributor to the generation of edema and hyperalgesia [24]. Moreover, neutrophils recruited to inflammatory foci are directly involved in the production of PGE_2_. Thus, in our study, reduction of zymosan-induced edema and hypernociception could be a result of reduced neutrophil recruitment to the knee joint leading to a reduced PGE_2_ release and/or of inhibition of pro-inflammatory mechanisms triggered by zymosan stimulation in the joint, including PAF, histamine, PGE_2_. The inhibition of neutrophil influx is associated with significant amelioration of overall disease in tissue [6,25,26,27]. Indeed, even though neutrophils are not involved in the perpetuation of chronic inflammation, they are thought to be key players in the context of articular inflammation. These cells secrete a wide array of toxic oxygen metabolites, proteolytic enzymes and inflammatory mediators that in turn lead to knee joint destruction and articular incapacitation [27]. In this context, it would be worthy to further investigate the effect of gedunin in cartilage degradation in a chronic model of articular inflammation. Our group has previously demonstrated that a pool of six different limonoids obtained from the seeds of *C. guianensis* (containing gedunin) presented a marked inhibition of cell influx and edema formation in a murine model of zymosan-induced arthritis [7]. Results presented here demonstrate that the *in vivo* treatment with gedunin greatly decreased neutrophil accumulation in mouse synovial washes and knee joint tissues after zymosan stimulation.

### 2.2. Gedunin Pre-Treatment Modulates Expression and Production of Inflammatory Mediators after Zymosan i.a. Stimulation

**Figure 2:**
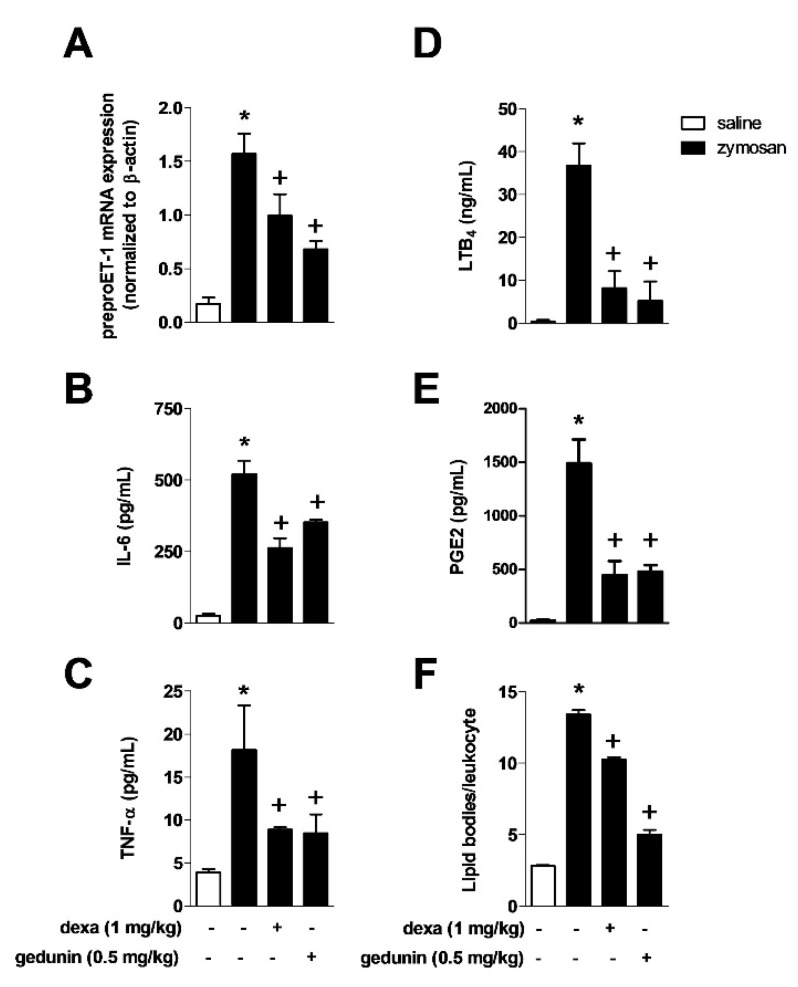
Pre-treatment with gedunin impairs zymosan-induced (**A**) ET-1 expression at 2 h and production of (**B**) IL-6, (**C**) TNF-α, (**D**) LTB_4_, (**E**) PGE_2_ and (**F**) the number of lipid bodies in synovial leukocytes 6 h after stimulation. Results are expressed as mean ± SEM. * *p* ≤ 0.05 *vs.* saline group, ^+^ *p* ≤ 0.05 *vs.* zymosan group.

Zymosan i.a. injection triggered increased production of several inflammatory mediators in knee joints 6 h after stimulation (Figure 2A,B,C and E). Mouse pre-treatment with gedunin (0.5 mg/kg) significantly reduced *in situ* expression of preproET-1 mRNA induced by i.a. stimulation with zymosan at 2h (Figure 2A), the production of IL-6 (Figure 2B), TNF-α (Figure 2C), LTB_4_ (Figure 2D) and PGE_2_ (Figure 2E) at 6h, in a similar extent as did dexamethasone. Six hours after i.a. injection of zymosan, it was observed a significant increase in the numbers of lipid bodies in synovial leukocytes, which are important sites for the synthesis and storage of lipid mediators and that increase in numbers during inflammatory responses.

Such a phenomenon was prevented by the *in vivo* pre-treatment with gedunin (0.5 mg/kg; i.p.) and dexamethasone (1 mg/kg; i.p.) and is correlated with diminished levels of lipid mediators in synovial washes.

ET-1 is a pro-inflammatory peptide that is produced by multiple cell types that induces neutrophil activation and chemotaxis [6,8], release of hydrogen peroxide [28] and inflammatory mediators [29]. The blockade of ET receptors [8] and the impairment of ET-1 production [6] are associated to reduced joint inflammation in zymosan-induced arthritis. Supporting these findings, we have previously demonstrated that i.a. injection of ET-1 induced knee joint swelling, neutrophil recruitment and TNF-α production in stimulated knee joints [6]. Here we demonstrate that the *in vivo* pretreatment with gedunin acutely inhibited zymosan-induced preproET-1 mRNA expression paralleled with decreased knee joint swelling, neutrophil recruitment, LTB_4_ and TNF-α production [30,31,32]. Numerous studies have highlighted the importance of LTB_4_ in the pathogenesis of RA. It has been demonstrated that 5LO^−/−^ mice failed to develop the collagen-induced arthritis [33] and showed a reduced number of recruited leukocytes in zymosan-induced inflamed joints [34]. LTB_4_ levels were increased in both synovial fluid [35] and serum [36] from RA patients, in addition to joints of mice submitted to models of monoarthritis, including zymosan-induced arthritis [6,8,29,37]. Additionally, neutrophil recruitment has been shown to depend on endogenous release of LTB_4_, since MK886 (a leukotriene synthesis inhibitor) reduced neutrophil migration to inflamed joints in mice with antigen-induced arthritis [38]. LTB_4_ has been shown to be involved in neutrophil-mediated zymosan-induced joint hypernociception [34]. In the present study, we observed that gedunin treatment decreased neutrophil influx to zymosan-injected knee joints as well as caused a marked suppression of zymosan-induced hyperalgesia. Even though hyperalgesia can also occur independently of neutrophil recruitment [29], previous reports demonstrate that neutrophil migration is correlated to inflammatory hypernociception [39,40,41,42,43,44], suggesting that the inhibition of neutrophil recruitment to inflamed joints by gedunin could contribute to reduced zymosan-induced hyperalgesia. Moreover, Guerrero and colleagues [34] have demonstrated that neutrophils produce LTB_4_ and PGE_2_, which are lipid mediators involved in hyperalgesia. In addition to that, the inhibition of TNF-α and IL-6 by gedunin also correlates with the amelioration of articular inflammation. Indeed, TNF-α plays a major role in articular inflammation by regulating other cytokines such as IL-6 and IL-8, as well as coordinating the recruitment of inflammatory cells into joints, including neutrophils and cartilage degradation [13,45]. In addition, anti-TNF therapy has been successful in clinical trials of arthritic patients, demonstrating solid efficacy in controlling signs and symptoms, with attenuation of arthritic parameters and retardment of joint damage [46,47,48]. The effects of IL-6 in experimental models of articular inflammation correlates with development of joint swelling and joint lesions [49,50,51]. The critical role for IL-6 in arthritis has also been evidenced by experimental models and clinical trials, in which blockade of IL-6 signaling diminished the signs and symptoms in experimental mice and rheumatoid arthritis patients that present resistance to TNF inhibition and to disease-modifying antirheumatic drugs [49,51,52,53,54,55].

### 2.3. Gedunin Impairs Neutrophil Activation in Vitro

**Figure 3:**
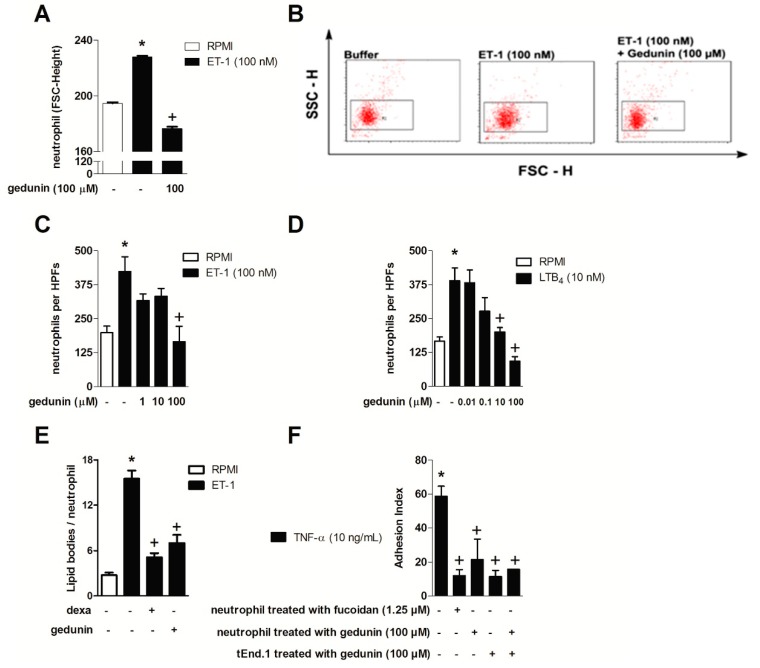
Gedunin *in vitro* blocks (**A**,**B**) neutrophil shape change; (**C**,**D**) chemotaxis; (**E**) lipid body formation and (**F**) adhesion. Results are expressed as mean ± SEM. * *p* ≤ 0.05 *vs.* non stimulated group, ^+^ *p* ≤ 0.05 *vs.* stimulated group.

The effect of gedunin on neutrophil activation and migration *in vitro* was assessed using ET-1 and LTB_4_ as stimuli, since neutrophils directly respond to these mediators via type A and B ET receptors and BLT1, undergoing shape change and chemotaxis [6,56,57]. As shown in Figure 3A,B, the pretreatment with gedunin (100 μM) impaired ET-1 (100 nM)-induced neutrophil shape change, as demonstrated by changes in forward scatter (FSC-H) (Figure 3B). In line with this result, neutrophil pretreatment with gedunin (100 μM) blocked ET-1-induced chemotaxis (Figure 3C). Stimulation of neutrophils with ET-1 also induced increased lipid body formation within 6 h, which was also impaired by gedunin pretreatment (Figure 3D). Moreover, gedunin pretreament inhibited LTB_4_-induced neutrophil chemotaxis (Figure 3E), starting at 10 μM, in a concentration dependent manner (R = 0.99). Supporting these results, the *in vitro* pretreatment of neutrophils with gedunin (100 μM) as well as with fucoidan (1.25 μM) impaired neutrophil adhesion to previously TNF-α-primed tEND.1 endothelial cells (Figure 3F). Interestingly, the pre-treatment of tEND.1 endothelial cells with gedunin 1 h prior TNF-α activation also reduced neutrophil adhesion, suggesting gedunin effect is not restricted to neutrophils. The pre-treatment of both neutrophils and tEND.1 cells (before adhesion and TNF-α priming, respectively) also reduced neutrophil adhesion index. It is important to note that incubation of neutrophils with gedunin did not cause cellular toxicity in doses ranging from 0.1 to 1000 μM, while higher doses (400 and 1000 μM) were cytotoxic (Table 1).

**Table 1:** Effect of gedunin on cell viability.

Sample	Dose	Viability (%)
Gedunin (μM)	1000	20
	400	40
	200	100
	100	100
	50	100
	20	100
	10	100
	1	100
	0.1	100
DMSO	50	100
	5	100
	0.5	100
Tween 2%	-	0

The inhibition of neutrophil shape change (which precedes leukocyte migration) triggered by ET-1 corroborated the direct effect of gedunin on neutrophils and is in line with the *in vivo* results in which gedunin treatment impaired neutrophil tissue accumulation. Therefore, the impairment of neutrophil accumulation achieved by gedunin treatment is likely to be a consequence of both a direct effect on these cells as well as of the diminished production of chemotatic mediators at inflamed site. Indeed, *in vitro* gedunin incubation also blocked ET-1- and LTB_4_-induced neutrophil chemotaxis, which supports the reduction in zymosan-induced neutrophil recruitment observed *in vivo*. Interestingly, in addition to gedunin effect on neutrophils, it is noteworthy that such limonoid also modulates tEnd.1 cell functions, as observed by diminished adhesion of neutrophils to tEnd.1 monolayers. The mechanisms by which gedunin reduces neutrophil attachment to endothelial cells remains to be investigated; however, it might modulates the expression of adhesion molecules or even the secretion of inflammatory mediators, including ET-1, as previously described by Toffoli and colleagues [58]. Reduced numbers of lipid bodies observed in gedunin-treated ET-1 stimulated leukocytes corroborates the direct capability of gedunin to modulate leukocyte functions. Lipid bodies are specialized cytoplasmic sites of compartmentalization of eicosanoid-forming enzymes with major roles in eicosanoid formation within inflammatory cells, and are key markers of leukocyte activation [59], being, therefore, a good target for treatment of inflammatory diseases.

### 2.4. Gedunin Post-Treatment Impairs Zymosan-Induced Edema Formation and Neutrophil Influx to Inflamed Knee Joints

**Figure 4:**
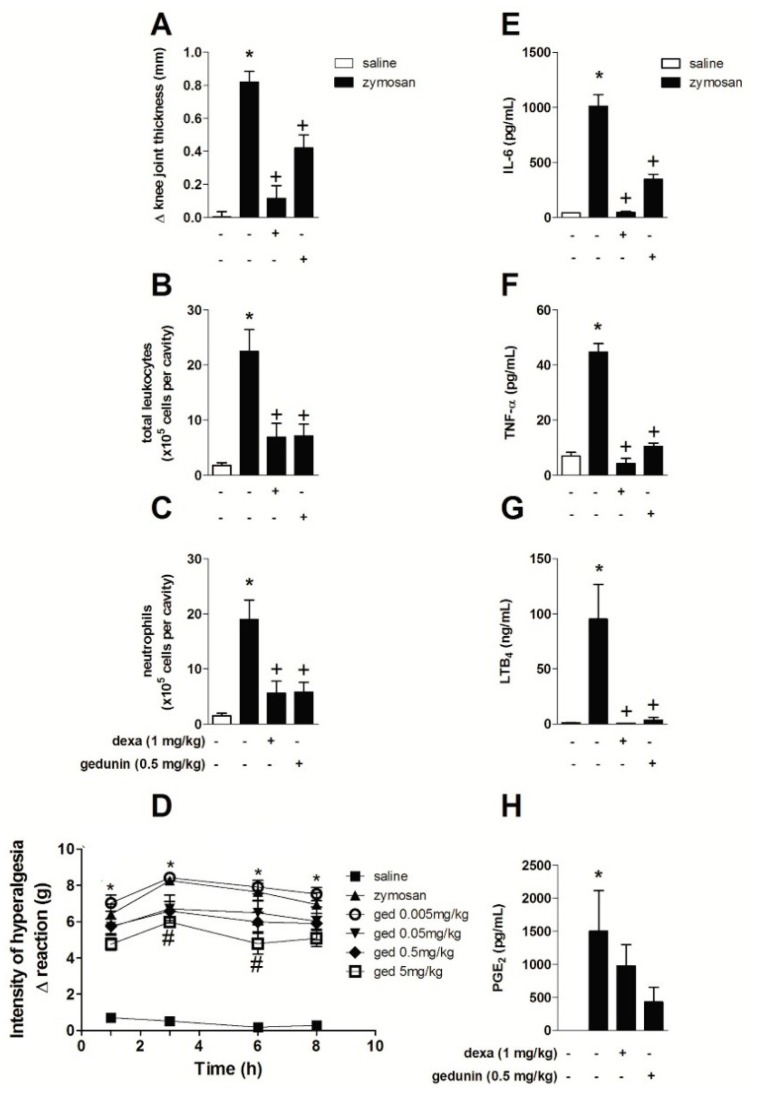
Gedunin post-treatments revert zymosan-induced (**A**) knee joint edema, (**B**) total leukocyte and (**C**) neutrophil influx, (**D**) hyperalgesia and production of (**E**) IL-6, (**F**) TNF-α, (**G**) LTB_4_, (**H**) PGE_2_ 10 h after stimulation. * *p* ≤ 0.05 *vs.* non stimulated group, ^+^ *p* ≤ 0.05 *vs.* stimulated group; ^#^ *p* ≤ 0.05 *vs.* zymosan group.

Similar to the results obtained with gedunin pretreatment, the post-treatment with gedunin (0.5 mg/kg; i.p.) 1 and 6 h after i.a. injection of zymosan (500 μg/cavity) also significantly reduced zymosan-induced edema formation (Figure 4A) and total leukocyte accumulation in synovial washes (Figure 4B), mainly due to the impairment of neutrophil influx into inflamed knee joints (Figure 4C) within 10 h. Post-treatment with dexamethasone also inhibited edema and leukocyte influx. The effect of gedunin post-treatment in the genesis of articular hypernociception during zymosan-induced arthritis was also evaluated. Gedunin i.p administration (5 mg/kg) 1 and 6 h after zymosan stimulation induced a significant antinociceptive effect within 5 and 8 h (Figure 4D), whereas no significant effect was observed with other doses in the time interval analyzed. Mouse post-treatment with gedunin also significantly reduced zymosan-induced IL-6 (Figure 4E), TNF-α (Figure 4F), LTB_4_ (Figure 4G) and PGE_2_ (Figure 4H) production when compared to zymosan-injected group. Similarly, dexamethasone post-treatment significantly impaired zymosan-induced LTB_4_, IL-6 and TNF-α production, whereas slightly diminished PGE_2_ levels.

Our data demonstrate that gedunin presents the ability to revert the inflammatory process, which might be partially explained by the induction of antiinflammatory or resolutive pathways. It is well known that dysfunctional resolution of inflammation is an underlying feature of chronic inflammatory conditions, including arthritis. However, whether gedunin triggers mechanisms of resolution of inflammation, such as apoptosis, clearance of activated inflammatory cells and reparative cytokines, deserves further investigation. Gedunin has been described as a modulator of Hsp90 activity [60], which triggers the activation of the transcription factor Heat Shock Factor-1 and increases the expression levels of Hsp70 [17,61]. Hsp70is a chaperone that presents antiinflammatory properties, as shown in several experimental models, including brain injury and myocardial ischemia [62,63]. Hsp70 has been shown to be expressed in rheumatoid arthritis synovial tissue and synovial fibroblast-like cells from patients with osteoarthritis and has been correlated with the amelioration of articular inflammation in patients with arthritis [64,65]. Collectively, these reports suggest a mechanism by which gedunin reverses zymosan-induced articular inflammation. In addition to that, Hsp90 has been reported to participate in the post-transcriptional control of cytokine production, including TNF-α [18]. This might be explained by the fact that nuclear factor related to kappa-B-binding protein is described as a client protein of Hsp90, which is in turn essential for the sustained NF-κB activity [66,67,68]. In line with this, we have previously demonstrated that gedunin impairs NF-kB nuclear translocation [21], which is known to be crucial for the expression of COX-2 and, therefore, limiting prostanoid production [69,70].

### 2.5. Gedunin Modulates Macrophage Activation in Vitro

**Figure 5:**
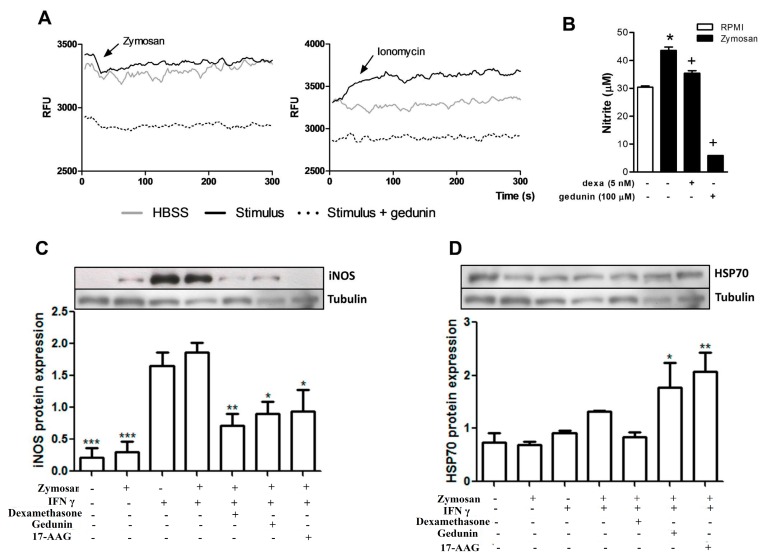
Gedunin *in vitro* treatment blocks (**A**) Ca^2+^ influx and also impairs (**B**) nitrite production and (**C**) iNOS and (**D**) Hsp70 expression in murine macrophages. Dexamethasone and 17-AAG were used as reference inhibitors. * *p* < 0.05 *vs* non-stimulated group, ^+^ *p* ≤ 0.05 *vs* stimulated group, ** *p* ≤ 0.01 *vs* stimulated group, *** *p* ≤ 0.001 *vs* non-stimulated group.

Macrophages are resident cells of the synovia that recognize zymosan stimulus via toll like receptor 2 (TLR2) and trigger the inflammatory response that occurs in the model of articular inflammation used in the present work [71]. We therefore evaluated the effect of gedunin pretreatment on macrophage activation *in vitro.* As shown in Figure 5A, the incubation of macrophages (5 × 10^4^/well) with zymosan (43 μg/mL) and with ionomycin (500 ng/mL, used as positive control) triggered a rapid calcium influx in macrophages, that was inhibited by the pre-incubation with gedunin (100 μM), demonstrating a direct effect of gedunin on the modulation of macrophage functions. Gedunin pretreatment also impaired NO production (Figure 5B) and iNOS expression (Figure 5C) induced by zymosan plus IFN-γ (100 U/mL) 24 h after stimulation. Since gedunin is described to inhibit the activation of the chaperone Hsp90, which is involved in TLR2 signaling pathway [17], 17-AAG (Hsp90 inhibitor) was used as positive control. Macrophage pretreatment with 17-AAG and dexamethasone also impaired iNOS expression by macrophages. It has been well described that Hsp90 inhibitors induce the expression of Hsp70 [20,22]. Therefore, we investigated whether gedunin induced anti-inflammatory mechanisms on macrophages, by evaluating its effect on Hsp70 expression by macrophages. Figure 5D demonstrates that the pre-incubation of macrophages with gedunin and 17-AAG induced Hsp70 expression in macrophages stimulated with zymosan plus IFN-γ, whereas dexamethasone incubation failed to change Hsp70 expression, when compared to control groups.

The combined *in vitro* and *in vivo* effects of gedunin reveal an important effect of gedunin as an antiarthritic candidate, specially due to its direct effect on key cells involved in articular inflammation, such as macrophages and neutrophils. The inhibition of iNOS expression and NO production by gedunin is an important anti-inflammatory effect for impairment of articular inflammation, since substantial evidence in the literature suggests that NO is implicated in articular inflammation and cartilage degradation in rheumatoid arthritis [72,73,74,75,76]. In an experimental model of osteoarthritis, the inhibition of Hsp90, which is one of the mechanisms of action of gedunin, increased Hsp70 protein levels in the knee joints, leading to protection of cartilage degradation and to suppression of macrophage activation [77], corroborating the results presented in this paper. The fact that diminished Hsp90 activity shifts the balance in favor of Hsp70 synthesis by macrophages might partially explain the antiinflammatory effects of gedunin observed *in vivo*.

## 3. Experimental Section

### 3.1. Animals

Male C57BL/6 mice (20 to 25 g) were obtained from the Oswaldo Cruz Foundation Breeding Unit (Fiocruz, Rio de Janeiro, Brazil). Mice were kept in plastic cages with free access to food and fresh water in a room with controlled temperature (22 to 24 °C) and light (12 h/12 h light/dark cycle) at the experimental animal facility until use. All experimental procedures were performed according to The Committee on Ethical Use of Laboratory Animals of Oswaldo Cruz Foundation (Fiocruz, #L62/12).

### 3.2. Induction of Acute Joint Inflammation

Acute joint inflammation was induced by intra-articular (i.a.) injection of zymosan (500 μg per cavity in 25 μL of sterile saline) by inserting a 27.5 G needle through the suprapatellar ligament into the left knee joint cavity, as previously described [8]. The contralateral knee joint was i.a. injected with the same volume of sterile saline solution, and was used as control. The analyses were performed 6 or 10 h after initial stimulation with zymosan.

### 3.3. In Vivo Pretreatments

Gedunin was intraperitoneally (i.p.) administered, in doses ranging from 0.005 to 0.5 mg/kg, in a final volume of 100 μL of sterile saline, 60 min before i.a. zymosan (500 μg per cavity) stimulation. Dexamethasone (1 mg/kg; i.p.), used as reference antiinflammatory drug, was also dissolved in sterile saline in a final volume of 100 µL per animal, 60 min before zymosan stimulation. Respective control groups were injected with the same volume of sterile saline solution. In hypernociception experiments, gedunin (0.005–5 mg/kg; i.p.) was administered 1 h prior intraplantar (i.pl.) zymosan (500 μg per cavity) stimulation. Evaluation of paw hypernociception was performed 2, 4 and 6 h after zymosan stimulation. The control group was injected with the same volume of sterile saline solution.

### 3.4. In Vivo Post-Treatments

Mice were treated with gedunin (0.5 mg/kg; i.p.), dexamethasone (1 mg/kg; i.p.) or saline solution, in a final volume of 100 µL, 1 and 6 h after i.a. injection of zymosan (500 μg per cavity). Evaluation of edema formation, total and differential counts were performed 10 h after zymosan stimulation. In hypernociception experiments, gedunin (0.005–5 mg/kg; i.p.) was administered 1 and 6 h after i.pl. zymosan (500 μg per cavity) stimulation. Evaluation of paw hypernociception was performed 3, 5, 8 and 10 h after zymosan stimulation. The control group was injected i.p. with the same volume of sterile saline solution.

### 3.5. Measurement of Knee Joint Swelling

Knee joint swelling was evaluated by measurement of the transverse diameters of the left knee joints using a digital caliper (Digmatic Caliper, Mitutoyo Corporation, Kanagawa, Japan). Values of knee joint thickness are expressed as the difference (Δ) between the diameter measured before (basal) and after induction of articular inflammation, in millimeters (mm).

### 3.6. Collection of Synovial Fluid and Leukocyte Counts

Mice were euthanized by CO_2_ inhalation at 6 or 10 h after i.a. injection of zymosan. Knee synovial cavities were washed with 300 μL of phosphate-buffered saline (PBS) containing EDTA (10 mM) by inserting a 21 G needle into the mouse knee joints and the synovial washes were recovered by aspiration. Total leukocyte counts were performed in a Neubauer chamber under an optical microscope, after dilution in Turk fluid (2% acetic acid). Neutrophil counts were performed using May-Grunwald-Giemsa-stained cytospins (Cytospin 3, Shandon Inc., Pittsburgh, PA, USA) under bright-field microscopes (1000× magnification) and values are expressed as numbers of cells per cavity (×10^5^). Cell-free synovial washes were stored at −80 °C for further analysis.

### 3.7. Histology

Whole knee joints obtained from C57BL/6 mice 6 h after i.a. administration of zymosan or saline were removed, dissected, and fixed in 10% formalin for 12 h. After decalcification in 10% EDTA in PBS solution for 1–2 weeks, the specimens were processed for paraffin embedding. Tissue sections (5 μm) were stained with hematoxylin and eosin (H&E) and mounted on permanent glass slides and analyzed under optic microscope (Olympus BX41, Olympus, Japan; original magnification, 200×).

### 3.8. Evaluation of Articular Hypernociception

Mechanical hypernociception was tested in mice as previously reported [34]. In a quiet room, mice were placed in acrylic cages (12 × 10 × 17 cm) with wire grid floors, 15–30 min before the start of testing. The test consisted of evoking a hind knee joint flexion reflex with a hand-held force transducer (electronic anesthesiometer; Insight, Ribeirão Preto, SP, Brazil) adapted with a 4.5 mm^2^ polypropylene tip. The investigator was trained to apply the tip perpendicularly to the central area of the hind paw with a gradual increase in pressure. The end point was characterized by the removal of the paw followed by flexion of knee joint. After the paw withdrawal, the intensity of the pressure was recorded automatically. The value for the response was an averaging of three measurements. The animals were tested before and after treatments. The results are expressed by delta (Δ) withdrawal threshold (in g) calculated by subtracting the zero-time mean measurements from the mean measurements at indicated time points after stimulus.

### 3.9. Real-Time RT–PCR for Evaluation of Preproet-1 Expression

Quantitative PCR (QPCR) was performed as previously described [78]. Briefly, mice were euthanized by CO_2_ inhalation, 2 h after i.a. injection of zymosan and knee joint complexes were harvested. Samples were homogenized in Trizol reagent and total RNA was extracted using the SV Total RNA Isolation System (Promega Biosciences LLC, San Luis Obispo, CA, USA). QPCR was performed in an ABI Prism 7000 Sequence Detection System using SYBR-green fluorescence (Applied Biosystems, Thermo Fisher Scientific, Inc., Waltham, MA, USA). The following primers were used: preproET-1, sense: 5'-TGT GTC TAC TTC TGC CAC CT-3', antisense: 5'-CAC CAG CTG CTG ATA GAT AC-3'; b-actin, sense: 5'-AGC TGC GTT TTA CAC CCT TT-3', anti-sense: 5'-AAG CCA TGC CAA TGT TGT CT-3'. The expression of β-actin mRNA was used as control for tissue integrity in all samples.

### 3.10. Determination of Inflammatory Mediators in Synovial Washes

Levels of TNF-α and IL-6 in cell-free knee joint washes were evaluated by commercial mouse inflammation Cytometric Bead Array system (CBA; BD Biosciences, Franklin Lakes, NJ, USA) using a FACScalibur flow cytometer (Becton and Dickinson, Franklin Lakes, NJ, USA), according the manufacturer’s instructions. LTB_4_ and PGE_2_ levels were evaluated in knee joint washes by enzyme immunosorbent assay (EIA) according to the manufacturer’s protocol (Cayman Chemical, Ann Arbor, MI, USA).

### 3.11. Lipid Body Induction and Staining

Lipid body evaluation was performed in leukocytes recovered from synovial cavities of zymosan-stimulated mice and in cultured mouse bone-marrow neutrophils (1 × 10^5^ cells) stimulated with ET-1 (100 nM) for 1 h (in 5% CO_2_ at 37 °C). Neutrophils were previously treated with gedunin (100 µM) or dexamethasone (5 nM) for 1 hour. While still moist, cells on cytospin slides were fixed in 3.7% formaldehyde in Ca^2+^/Mg^2+^-free HBSS pH 7.4 rinsed in 0.1 M cacodylate buffer 1.5% OsO_4_ for 30 min, rinsed in dH_2_O, immersed in 1.0% thiocarbohydazide for 5 min, rinsed in 0.1 M cacodylate buffer, restained in 1.5% OsO_4_ for 3 min, rinsed in dH_2_O, and then dried and mounted. The morphology of fixed cells was observed, and lipid bodies were enumerated by light microscopy with a 100× objective lens in 50 consecutively scanned leukocytes.

### 3.12. Shape Change Assay

Human peripheral polymorphonuclear leucocytes (PMNLs) were obtained by venipuncture, from male adult healthy volunteers (25–30 years; with full consent, according to The Research Ethics Committee of Fiocruz/CEP, license #346.627, Rio de Janeiro, Brazil), who had taken no systemic medication for at least 72 h before donating blood. Neutrophils were isolated and purified by dextran sedimentation followed by Percoll discontinuous gradients (>90% final purity). Aliquots of 5 × 10^5^ neutrophils were incubated in the presence of ET-1 (100 nM) or RPMI in a 37 °C shaking water bath for 6 min, after which the reaction was stopped by the addition of a paraformaldehyde solution (0.25%) at 4 °C and placed on ice until analysis. To evaluate the effect of gedunin on ET-1-induced shape change, samples were pre-incubated with gedunin (100 nM) for 15 min (37 °C) before ET-1 stimulation. Samples were immediately analyzed on a FACScalibur flow cytometer (Becton Dickinson, Franklin Lakes, NJ, USA). Acquisition was set using the FL-2 fluorescence channel, through which human neutrophils can be distinguished from eosinophils by means of their auto-fluorescence characteristics. Five hundred neutrophils were acquired for each of the triplicate samples. As measurement of shape change, data are reported as the change in FSC-H compared with buffer-treated cells.

### 3.13. Neutrophil Chemotaxis

Neutrophils isolated from C57BL/6 mouse bone marrow were purified by Percoll discontinuous gradients, washed and resuspended in RPMI 1640 supplemented with 10% FBS and then assayed in a 48-well microchemotaxis Boyden chamber (Neuroprobe Inc., Gaithersburg, MD, USA). The bottom wells of the chamber were filled with 28 µL of LTB_4_ (10 nM), ET-1 (100 nM) or RPMI 1640 (control), whereas the upper wells (separated with a 3 mm polycarbonate filter; Nuclepore, Sigma Aldrich, St. Louis, MO, USA) were filled with neutrophils (10^5^ cells; 50 µL) that had been previously incubated with gedunin (0.01–100 µM) for 30 min. The chamber was incubated in humidified air with a 5% CO_2_ atmosphere at 37 °C for 60 min. Cells that migrated through the filter were counted under light microscopy. Neutrophil chemotaxis was calculated and expressed as the mean number of migrated cells in five random high-power fields per well (in quadruplicate).

### 3.14. Cell Adhesion Assay

The murine vascular endothelial cell line tEnd.1 was cultured in RPMI 1640 medium supplemented with 10% FBS, 2 mM l-glutamine, 100 IU/mL penicillin, and 100 μg/mL streptomycin (37 °C, 5% CO_2_). tEnd.1 cells were plated onto 24-well culture chambers (10^4^ cells per well; Nunc, Thermo Fisher Scientific, Inc., Waltham, MA, USA) for 24 h. Before each experiment, tEnd.1 cells were treated for 4 h with recombinant mouse TNF-α (10 ng/mL). Neutrophils isolated from bone marrow of naïve C57BL/6 mice were pre-treated for 1 h with fucoidan (1.25 μM) or gedunin (50 μM). The pre-treated neutrophils were then allowed to adhere to tEnd.1 cultures (50 neutrophils per tEnd.1) for 1 h. Non-adherent cells were gently washed out with PBS and the remaining cells were subsequently fixed in ethanol, stained with Giemsa (Merck, Darmstadt, Hessen, Germany) and analyzed under an inverted microscope (Olympus CK40, Olympus Corporation, Tokyo, Japan). The number of adherent neutrophils per tEnd.1 cell was determined by direct counting. Data are expressed as an association index which was calculated as follows: Index of adhesion = (tEnd.1 with bound leukocytes) × (total tEnd.1 number)^−1^ × (neutrophils bound to tEnd.1) × (total tEnd.1 number)^−1^ × 100, as previously described [79].

### 3.15. Cytotoxicity Assay

Cytotoxicity of gedunin was determined in neutrophils recovered from C57BL/6 mouse bone marrows. Viable cells were seeded in a flat bottom 96-well plate (2 × 10^6^ cells/well, in quadruplicate) and cultured for 1 h (in 5% CO_2_ at 37 °C). The cells were cultured in the presence of different concentrations of gedunin at 0.1–1000 μM for 20 h, after which were incubated with MTT [3-(4,5-dimethylthiazol-2-yl)-2,5-diphenyltetrazolium bromide] solution (5 mg/mL of saline; 22.5 mL per well) for 4 h. The supernatant was discarded and dimethyl sulfoxide (DMSO; 150 μL per well) was added for formazan crystal solubilization (Spectramax M5, Molecular Devices, Sunnyvale, CA, USA). The absorbance was read at 540 nm. The concentrations of gedunin that induced ≥ 10% of cell death were considered cytotoxic, and were not used in the biological assays.

### 3.16. Calcium Mobilization Assay

Intracellular calcium influx ([Ca^2+^]i) was measured using the FLIPR Calcium Plus Assay kit on FlexStation II (Molecular Devices, Sunnyvale, CA, USA) fluorometric microplate reader, according to the manufacturer’s instructions. Briefly, gedunin (100 μM) pretreated J774 macrophages (5 × 10^4^/well) were exposed to ionomycin (500 ng/mL) or zymosan (43 μg/mL). Fluorescence intensity ratios at 485/525 nm (λ_ex_/λ_em_) were recorded up to 5 min and analyzed through SoftMax Pro software (Molecular Devices, Sunnyvale, CA, USA).

### 3.17. Determination of Nitrite Production

Peritoneal macrophages recovered from C57BL/6 mice, treated or not with gedunin (100 uM) or dexamethasone (5 nM) for 1 h, were plated onto 96-well sterile flat-bottomed tissue culture plates in a final concentration of 1 × 10^6^ cells/well (RPMI 1640 medium supplemented with 10% FBS, in 5% CO_2_ at 37 °C). Macrophages were stimulated with zymosan (10 μg/mL) plus IFN-γ (100 U/mL) for 24 h, after which supernatants were harvested for nitrite determination. Nitrite levels were determined by the addition of 100 μL modified Griess reagent (Sigma Aldrich, St. Louis, MO, USA) to the wells for 15 min at room temperature. Absorbance was read at 562 nm using a Spectramax M5 microplate reader (Molecular Devices, Sunnyvale, CA, USA). The concentration of nitrite was calculated from a sodium nitrite standard curve (range 1.5–100 μM).

### 3.18. Western Blotting

J774A.1 cells were (1 × 10^5^ cells) pretreated with gedunin (100 μM) or dexamethasone (1 nM) or 17-AAG (Hsp90 inhibitor, 0.3 µM) for 1 h and stimulated with zymosan (43 μg/mL) were lysed in buffer containing protease inhibitors. Twenty µg of protein extract were resolved by SDS-PAGE, and proteins were transferred to a polyvinylidenedifluoride (PVDF) membrane. Membranes were probed with antibodies (Santa Cruz Biotechnology, Dallas, TX, USA) against nitric oxide synthase 2 (NOS2), Hsp70 and α-tubulin, which were used as control for protein gel loading. Antibodies were diluted in Tris-buffered saline containing 0.5% Tween 20 and 5% nonfat dry milk, and incubated overnight at 4 °C. On the following day, the membranes were incubated with horseradish peroxidase-conjugated secondary IgG (Santa Cruz Biotechnology) for 1 h at room temperature. The blots were developed with the use of a chemiluminescent substrate (ECL Western blotting Analysis System; Amersham Biosciences, Piscataway, NJ, USA).

### 3.19. Statistical Analysis

Results are reported as the mean ± SEM and were statistically analyzed by means of analysis of variance (ANOVA) followed by Newman-Keuls-Student test or the Student’s t test. Values of *p* ≤ 0.05 were regarded as significant.

### 3.20. Drugs and Reagents

Phosphate-buffered saline (PBS), Tween-20, fucoidan, ethylenedyaminetetracetic sodium salt (EDTA), percoll, sodium citrate, HEPES, bovine serum albumin (BSA), phosphate citrate buffer, Hank’s balanced salt solution, ET-1 and dexamethasone were purchased from Sigma Chemical Co. (St Louis, MO, USA). TNF-α and KC/CXCL1 matched antibody pairs were obtained from R&D Systems (Minneapolis, MN, USA). Fetal bovine serum (FBS) was obtained from HyClone (Logan, UT, USA). LXA4 [5(*S*),6(*R*),15(*S*)-trihydroxyeicosa-7-*trans*-9-*trans*-11-*cis*-13-transtetraenoic acid] and LTB_4_ were purchased from Cayman Chemical Company. Recombinant TNF-α was obtained from R&D Systems. Endotoxin-free gedunin was purchased from Gaia Chemical Corporation (Gaylordsville, CT, USA). Gedunin (1(*S*),3a(*S*),4a(*R*),4b(*S*),5(*R*),6a(*R*),10a(*R*),10b(*R*),12a(*S*)-5-(acetyloxy)-1-(3-furanyl)-1,5,6,6a,7,10a,10b,11,12,12a,decahydro-4b,7,7,10a,12a-pentamethyloxireno[c]phenanthro[1,2-d]-pyran-3,8(3a*H*,4b*H*)-dione) purity (>95%) was certified by thin layer chromatography by Gaia Chemical Co. and by high-speed countercurrent chromatography (HSCC) (performed on a model HSCCC-1000, Pharma-Tech Research Corp., Baltimore, MD, USA) at Farmanguinhos Natural Product Laboratory (Rio de Janeiro, Brazil).

## 4. Conclusions

In the present study, we demonstrated that gedunin, a natural tetranortriterpenoid from the Meliaceae family, possesses anti-inflammatory and analgesic activities. *In vivo* pre- and post-treatment with gedunin impaired several features of murine zymosan-induced arthritis in mice, including knee joint swelling, neutrophil influx, hyperalgesia and production of inflammatory mediators. Moreover, we demonstrate that gedunin directly impairs neutrophil and macrophage activation by impairing calcium influx, cell adhesion, chemotaxis and lipid body formation.
